# Unpicking the Semantic Impairment in Alzheimer’s Disease: Qualitative Changes with Disease Severity

**DOI:** 10.3233/BEN-2012-0346

**Published:** 2011-12-29

**Authors:** Faye Corbett, Elizabeth Jefferies, Alistair Burns, Matthew A. Lambon Ralph

**Affiliations:** ^1^University of ManchesterManchesterUK; ^2^University of YorkYorkUK; ^3^Neuroscience and Aphasia Research Unit (NARU)School of Psychological SciencesZochonis BuildingUniversity of ManchesterManchesterUK

**Keywords:** Alzheimer's disease, semantic, severity, access, storage

## Abstract

Despite a vast literature examining semantic impairment in Alzheimer's disease (AD), consensus regarding the nature of the deficit remains elusive. We re-considered this issue in the context of a framework that assumes semantic cognition can break down in two ways: (1) core semantic representations can degrade or (2) cognitive control mechanisms can become impaired [1]. We hypothesised and confirmed that the nature of semantic impairment in AD changes with disease severity. Patients at mild or severe stages of the disorder exhibited impairment across various semantic tasks but the nature of those deficits differed qualitatively for the two groups. Commensurate with early dysfunction of the cognitive control, temporoparietal-frontal-cingulate network, characteristics of deregulated semantic cognition were exhibited by the mild AD cases. In contrast, the severe AD group reproduced features of additional degradation of core semantic representations. These results suggest that spread of pathology into lateral anterior temporal lobes in later stage AD produces degradation of semantic representations, exacerbating the already deregulated system. Moreover, the dual nature of severe patients’ impairment was highlighted by disproportionately poor performance on tasks placing high demand on both conceptual knowledge and control processes–e.g., category fluency.

## 1. Introduction

Semantic cognition encompasses the processes and representations that support the storage and controlled use of our everyday factual knowledge, including the meanings of words, pictures, objects, faces, sounds and events. It provides the foundation for almost everything we do and so it is no surprise that a deficit in this domain can have a highly debilitating effect on patients' daily lives. Direct comparisons of different semantically-impaired neurological populations have allowed us to examine the neural network underpinning conceptual knowledge in detail [1–3]. These studies have shown that core semantic cognition is supported by at least two interacting principal components: (1) a repository of semantic knowledge formed through a unified distillation of modality-specific sources of sensorimotor and verbal information; and (2) an executive control component responsible for shaping or controlling semantic activation in a time, task and context appropriate fashion. Consistent with this hypothesis, a few direct comparisons of different neurological groups have shown that damage to these different aspects of semantic cognition lead to qualitatively distinct impairments [1,4].

Alongside profound episodic memory impairment, patients with Alzheimer's disease (AD) exhibit notable deficits in their semantic performance, even at the very

ISSN 0953-4180/12/$27.50 © 2012 – IOS Press and the authors. All rights reserved early stages of the disorder [5–7]. Although there is a vast literature examining semantic memory in AD, no consensus regarding the nature of the deficit has yet been reached. Two possible causes have been identified; on the one hand, AD has been characterised as a 'storage' disorder in which representations are lost from semantic memory. Proponents of this view report a pattern of gradual decline of knowledge in AD, leading to a consistent impairment of the same concepts across different tasks [8–13]. Alternatively, others have shown that the level of semantic impairment in this condition is not stable but varies in line with changing task demands. Performance on semantically-demanding lexical and conceptual decision tasks, for example, has been shown to improve significantly with the provision of constraining cues [14–17]. This finding is not consistent with the notion that knowledge has been permanently lost in AD but can been explained in terms of a problem with accessing that information. There are some hints in the literature that the disparity between these viewpoints could reflect a severity-related difference. For example, while early AD patients' level of semantic knowledge seems to vary in line with executive difficulty [17], the same is not true of more severely-impaired patients who exhibit greater consistency across different tasks [18]. The relationship between severity and the nature of semantic impairment in AD has not, however, been examined directly in previous studies. It seems likely that gradual spreading of pathology over the course of AD will compromise separable components of the semantic network at different times. This view anticipates that the qualitative nature of semantic impairment in AD will change throughout the disease. By taking severity into account, therefore, it might be possible to resolve the longstanding conundrum regarding the nature of semantic impairment in this disorder.

We have previously employed a case-series, comparative design to inspect semantic impairments arising in different neurological groups (e.g., HSVE vs. semantic dementia (SD): [4]; semantic aphasia (SA) vs. SD: [1].) SD is a neurodegenerative disorder characterised by a selective semantic deficit affecting knowledge across a full range of modalities in both expressive and receptive tasks [19–23]. This condition occurs in the context of atrophy focussed on the ATL bilaterally [24, 25]. Multimodal semantic deficits are also observed in some patients following a stroke [1–3]. In contrast to SD, however, patients with SA have damage to left prefrontal and/or temporoparietal regions with complete sparing of the ATL (see [3]). In a direct comparison, Jefferies and Lambon Ralph [1] found that these two groups failed semantic tasks for different reasons. The ATL atrophy in SD was associated with a pattern of impairment indicative of degraded semantic representations and their semantic performance was impervious to changes in task demand and modality. Greatest impairment was observed for the least familiar/low frequency concepts suggesting that these items were most vulnerable to degradation. In contrast, the SA patients' performance suggested that conceptual knowledge was not degraded in these cases, but a failure of regulatory cognitive control underpinned their impaired semantic cognition. The SA patients' performance was distinct in the following key ways: a) they were much less consistent across tasks and strongly influenced by task demands; b) in addition to coordinate and superordinate naming errors (e.g., squirrel → "rabbit" or "animal"), the SA patients produced associative errors (e.g., squirrel → "nuts") which were almost never observed in SD; (c) naming and comprehension scores were improved or diminished by the provision of cues or other forms of task constraint (which had little or no effect in SD); (d) the SA patients exhibited semantic "access" and refractory symptoms in contrast to the "storage" deficits of SD [26–28] and (e) their performance on comprehension tasks was predicted by the control requirements within an individual trial. These findings are consistent with the view that two interacting components support semantic cognition: 1) a modality-invariant, representational hub supported by the ATL bilaterally [29–32] and, 2) an executive control component underpinned by a network encompassing the left prefrontal cortex and regions in the temporoparietal cortex [1–3,33]. Formal meta-analyses of functional neuroimaging in normal participants indicate that, in addition to these two regions, cingulate cortex also contributes to semantic cognition [34]. As noted below, these three areas are implicated in the earliest phases of AD.

A considerable volume of research has been dedicated to tracing disease pathogenesis over the course of AD. These studies indicate that the functionally separable components of semantic cognition are likely to become compromised at different stages of the disease. In the earliest stages of AD, amyloid plaques and neurofibrillary tangles occupy the medial temporal lobe (MTL) – a critical region supporting episodic memory [35–37]. The broader MTL network incorporating, amongst other regions, the posterior cingulate cortex is also found to be hypometabolic at this early stage of the disease, with probable impact on episodic memory as well as executive control functions [38–40]. Beyond

Table 1
Performance on attentional/executive and semantic tasks

| Task | Max | Control mean (s.d.) | SA mean | Mild AD mean | Severe AD mean | SD mean |
|---|---|---|---|---|---|---|
| *Executive/ attentional* | | | | | | |
| WCST (categories) | 6 | 1* | 1.2 | 2.1 | 0.4 | NT |
| Elevator counting | 7 | 6* | 5 | 6 | 4 | NT |
| Elevator counting (with distraction) | 10 | 2*‡ | 2 | 5 | 1 | NT |
| Ravens' Matrices (percentiles) | – | – | 4/10 cases = 50, remaining cases < 10 | 4/10 cases = 25, 1 case = 50, remaining cases ⩾ 80 | 7/10 cases = 5, remaining cases = 10 | 9/10 cases > 90, remaining case = 75 |
| *Semantic* | | | | | | |
| PPT picture | 52 | 51.2 (1.4) | 40 | 42 | 34 | 41 |
| PPT word | 52 | 51.1 (1.1) | 41 | 45 | 35 | 39 |
| 64 item battery | | | | | | |
| Naming | 64 | 62.3 (1.6) | 21 | 49 | 40 | 27 |
| WPM | 64 | 63.7 (0.5) | 50 | 57 | 46 | 46 |
| CCT picture | 64 | 58.9 (3.1) | 36 | 38 | 26 | 40 |
| CCT word | 64 | 60.7 (2.06) | 37 | 46 | 33 | 37 |
| Environmental sounds | | | | | | |
| Spoken word – picture | 48 | 47.8 (0.6) | 39 | 40 | 30 | 33 |
| Sound – picture | 48 | 41.2 (2.5) | 28 | 19 | 11 | 22 |
| Sound – word | 48 | 40.8 (3.8) | 25 | 17 | 11 | 20 |
| Category fluency (6) | – | 95.7 (16.5) | 14 | 37 | 10 | 31 |
| Letter fluency | – | 44.2 (11.2) | 5 | 26 | 8 | 21 |

*denotes normal cut-off; NT = not tested. ‡normal cut-off for participants aged 65–80.

the MTL, early AD neuropathology critically undermines the frontal-temporoparietal control network in two key ways: 1) cells within the superior longitudinal fasciculus are selectively affected causing disconnection of frontal and temporoparietal regions [3]; and 2) spreading of pathology into the basal forebrain, particularly the basal nucleus of Meynert, undermines the cholinergic system that normally provides critical innervation to areas of the neocortex supporting executive control, including prefrontal and parietal lobes as well as the thalamus [41]. Consistent with these changes, patients in the early stages of AD have executive and attentional deficits [42–45]. As the disease reaches more moderate stages, generalised hypometabolism of the temporal lobe (TL) is joined by plaques and tangles invading inferolateral aspects of this region [7,46] – notably the same areas that underlie the semantic impairment in SD [24,25,47,48].

The present study examined the hypothesis that the evolution of pathology in AD generates qualitatively different semantic impairments over the course of the disease. In the early stages, damage to the frontal-temporoparietal-cingulate control network should produce deregulation of semantic cognition. As neuropathology spreads into the inferolateral aspects of the ATL, in the more severe phase of AD, semantic representations themselves will begin to degrade, thus exacerbating the already deregulated semantic system. Assuming this hypothesis is true, early AD cases should exhibit semantic impairment that is characteristically similar to that observed in SA, while additional traits typical of SD should emerge when the disease reaches a later stage. We tested this hypothesis by extending Jefferies and Lambon Ralph's [1] case-series, comparative design to include patients with AD. In doing so we were able to compare the nature of semantic impairment at mild and severe stages of AD against these two "reference" groups.

## 2. Method

### 2.1. Participants

This work was approved by local health authority ethics committees and informed consent was obtained for all participants. Twenty patients with Alzheimer's disease ($n = 10$ mild, $n = 10$ severe) were recruited from dementia services in Manchester, UK. All cases were diagnosed by an experienced Old Age Psychiatrist (AB) with respect to the standard, international consensus criteria. Patients with a history of stroke or other neurological conditions were excluded. All participants in the mild AD group scored 19 or more (out of 30) on the Mini-Mental State Examination (MMSE [49]), whereas all severe cases obtained 14 or less (mean score: mild = 21.3; severe = 10.1; $t(18) > 8.82$, $p < 0.001$, see Table 1). The mild group were numerically but not significantly older than the severe group. The two AD groups were compared with the SA and SD patient groups previously reported by Jefferies and Lambon Ralph [1] and Bozeat et al. [50]. The ten SA patients were recruited from stroke clubs and speech and language therapy services in the Manchester area. SA patients were identified on the basis that they failed both word and picture versions of the Camel and Cactus semantic association task [50]. Every case had chronic impairment from a stroke at least a year prior to testing. All patients had damage to the left prefrontal cortex and/or areas in the temporoparietal region. The second comparative group comprised ten patients with SD who were identified through the Memory and Cognitive Disorders clinic at Addenbrooke's Hospital, Cambridge. These patients met all published criteria for SD [51]; they had word finding difficulties despite fluent speech and showed impaired semantic knowledge and single word comprehension but phonology, syntax, visuo-spatial abilities and day-to-day memory remained relatively well preserved [50]. Focal bilateral atrophy of the infero-lateral aspects of the anterior temporal lobe was identified in every case using MRI.

### 2.2. Assessments

#### 2.2.1. Attentional/executive

All four groups were assessed on the Raven's coloured progressive matrices test of non-verbal reasoning [52]. In addition, the AD and SA groups' attentional/executive skill was assessed with the Wisconsin Card Sorting Test [53] and the Elevator Counting task with and without distraction (taken from the Test of Everyday Attention [54]).

#### 2.2.2. Semantic memory assessment

A range of tests were used to assess semantic cognition. The Pyramids and Palm Trees test examines semantic associative knowledge (PPT [55]). This task requires participants to decide which of two response options (e.g., palm tree/pine tree) is most closely related to the probe item (e.g., pyramid). The task was assessed in both picture and word formats. Fluency was examined for six categories (animals, birds, fruit, household items, tools and vehicles). Patients were given one minute to produce as many members of each category as possible. Category fluency was compared to performance on a letter fluency task (participants had to produce as many words beginning with the letters F/A/S).

Three tasks from the Cambridge semantic battery were used to assess knowledge of sixty-four concrete concepts drawn from the same six categories listed above. The tasks were:

i) The Camel and Cactus test (CCT [50]): this is a semantic association task similar to the PPT. Participants are required to select which of four response options is most related to a probe item. The test was administered in both word and picture formats.
ii) Spoken word-to-picture matching: participants are given the name of an item and asked to select the corresponding picture. Ten picture stimuli (the target and nine foils) drawn from the same semantic category were presented on each trial.
iii) Picture naming: patients were presented with a line drawing of each item in the battery ($n = 64$) and asked to name it.

The environmental sound battery [50] employs characteristic sounds to assess knowledge relating to 48 items drawn from six categories (domestic/foreign animals, human sounds, household items, vehicles and musical instruments). The battery requires the participants to match a probe to a target within an array of eight possible within-category alternatives. Three identity-matching conditions were assessed: sound-picture, sound-word and spoken word-picture matching.

Two tests were used to examine the effect of cueing on picture naming in AD.

(a) Cued and miscued naming: Patients were asked to name the 60 pictures from the Boston Naming Test [56]. Each item was assessed under cued, miscued and uncued conditions on separate occasions. A cue consisted of the first two phonemes of the target response whereas the phonemes of a semantic competitor were provided on the miscued trials. (b) Progressive phonological cueing: participants were asked to name thirty items selected from the Snodgrass and Vanderwart [57] stimulus set. If an item was not named spontaneously, then the first phoneme of the target response was provided as a cue. Cues increased in length, one phoneme at a time, until either the item had been named correctly, or the final phoneme had been provided.

## 3. Results

### 3.1. Background neuropsychological examination

#### 3.1.1. Attentional/executive

Jefferies and Lambon Ralph [1] found that SA patients performed poorly across a range of attentional/executive tasks (see Table 1 for mean scores). Conversely, patients with SD rarely show a deficit on this type of assessment. All SD cases re-reported in the present study, for example, scored within the top 25th percentile of the Raven's coloured progressive matrices test.

We examined the AD patients on a number of attentional/executive measures previously used to examine the SA patients. In line with previous assessments of this domain in early AD [42,44], the mild AD group exhibited some evidence of impairment on these tasks while the severely impaired AD patients attained scores below the normal level on all attentional/executive measures (see Table 1 for mean scores and Table 1a for individual AD patient data). The two groups performed equivalently on the WCST; 6/10 mildly impaired cases and 7/10 severely impaired patients did not complete a single category ($t$ (18) = 1.83, n.s). The elevator counting task (without distraction) was passed by 9/10 cases in the mild group compared to only 5/10 patients in the severe group but the groups' overall scores did not differ significantly ($t$ (11) = 2.06, n.s.). When elevator counting was examined with distraction a significant group difference was observed with only 3/10 mild cases scoring below the normal cut-off compared to 7/10 patients in the severe group ($t$ (13) = 2.53, $p$ = 0.026). Significantly better performance in the mild group was also observed for the Raven's progressive matrices task ($t$ (9) = 5.21, $p < 0.001$).

#### 3.1.2. Semantic

All groups exhibited impairment across a full range of picture/word and sound-based tasks suggesting a multimodal semantic impairment in every case (see Tables 1 and 1a). The severity of semantic impairment was largely equivalent for the SA and SD groups whose scores only differed significantly on two tasks: letter fluency (SD > SA: $t$ (17) = 4.3, $p < 0.001$) and sound-picture matching (SA>SD: $t$ (18) = 2.2, $p = 0.04$). The severely impaired AD group performed substantially worse than the mild group on all tasks in the semantic battery ($t$(15–18) > 2.45, $p < 0.018$). Relative to the SA patients, the mild AD group obtained substantially higher scores on four semantic tasks (naming, word CCT, category fluency and letter fluency: $t$ (12–18) > 2.31, $p < 0.03$) but significantly poorer scores on two of the environmental sounds tasks (sound-picture/word matching: $t$ (18) > 2.75, $p < 0.01$). The SD group were substantially worse than the mild AD group in picture naming ($t$ (11) = 3.11, $p = 0.01$). In contrast, the severe AD group obtained significantly lower scores than the SA group on seven semantic assessments (naming, picture/word PPT, picture CCT and all three subtasks from the environmental sounds battery: $t$ (11–18) > 2.06, $p < 0.05$) and were worse than the SD patients for five tasks (picture CCT, category fluency, letter fluency, sound-picture matching and sound-word matching: $t$ (12–17) > 2.33, $p < 0.04$).

### 3.2. Examining the nature of semantic impairment

In their case-series comparative study, Jefferies and Lambon Ralph [1] identified a number of ways in which semantic impairments due to a failure of regulatory control (SA) could be distinguished from those due to degraded semantic representations (SD). The same analyses were employed in the present study to examine the nature of semantic impairment at mild and severe stages of AD. We hypothesised that a failure of regulatory control processes in the early stage of AD would lead to deregulated semantic cognition. Specifically, we expected mild cases to exhibit the following three characteristics of semantic deregulation previously observed in SA: 1) an association between performance on semantic tasks and attentional/executive assessments; 2) a strong relationship between the ability to make semantic associative judgements in the CCT and the degree of conceptual control required by a particular trial; and 3) naming accuracy can be both improved (by the provision of correct cues) and diminished (by miscues). Due to the progressive nature of AD, however, it was expected that a qualitatively different semantic impairment would emerge later in the disorder as semantic deregulation was joined by gradual degradation of semantic representations. The impact of this dual impairment would be expected to be especially pronounced on tasks that place a large demand on both conceptual knowledge and semantic control, such as category fluency. Furthermore, relative to mild AD patients, severe cases should begin to show characteristics of a storage problem as exhibited by SD patients, including: 1) more consistent impairment of conceptual knowledge across different semantic tasks; and 2) a stronger influence of conceptual familiarity/frequency on performance. Given that we have formally demon-

Table 1a,
Individual AD patient data

| Task | Max | Control | Mild AD | | | | | | | | | | Severe AD | | | | | | | | | |
|---|---|---|---|---|---|---|---|---|---|---|---|---|---|---|---|---|---|---|---|---|---|---|
| | | mean (s.d.) | EM$^1$ | EM$^2$ | LL | MH | DS | MO | JM$^1$ | DM | JH | TA | EA | JL | ER | BL | AD | LA | ML | EB | JM$^2$ | AW |
| *Executive/ attentional* | | | | | | | | | | | | | | | | | | | | | | |
| WCST (categories) | 6 | 1* | 6 | 6 | 3 | 0 | 0 | 0 | 0 | 0 | 0 | 6 | 0 | 1 | 0 | 0 | 0 | 0 | 0 | 0 | 2 | 1 |
| Ravens' Matrices (percentiles) | – | – | 95 | 25 | 90 | 50 | 82.5 | 80 | 25 | 92.5 | 25 | 25 | 10 | 5 | 5 | 5 | 5 | 10 | 5 | 5 | 5 | 10 |
| Elevator counting | 7 | 6* | 7 | 6 | 7 | 4 | 6 | 7 | 6 | 7 | 6 | 7 | 6 | 2 | 0 | 3 | 1 | 7 | 7 | 7 | 4 | 7 |
| Elevator counting | 10 | 3* | 10 | 3 | 6 | 0 | 1 | 4 | 4 | 9 | 0 | 8 | 2 | 1 | 1 | 1 | 0 | 0 | 5 | 3 | 0 | 0 |
| *Semantic* | | | | | | | | | | | | | | | | | | | | | | |
| (with distraction) | | | | | | | | | | | | | | | | | | | | | | |
| PPT picture | 52 | 51.2 (1.4) | 44 | 43 | 52 | 32 | 47 | 37 | 40 | 46 | 42 | 40 | 29 | 42 | 25 | 26 | 32 | 32 | 45 | 43 | 25 | 36 |
| PPT word | 52 | 51.1 (1.1) | 45 | 47 | 49 | 34 | 46 | 40 | 46 | 46 | 45 | 49 | 39 | 42 | 24 | 26 | 30 | 39 | 41 | 46 | 27 | 33 |
| 64 item battery | | | | | | | | | | | | | | | | | | | | | | |
| Naming | 64 | 62.3 (1.6) | 49 | 42 | 61 | 44 | 49 | 45 | 57 | 49 | 57 | 39 | 52 | 48 | 39 | 39 | 36 | 42 | 37 | 29 | 41 | 32 |
| WPM | 64 | 63.7 (0.5) | 63 | 50 | 64 | 40 | 57 | 58 | 61 | 56 | 61 | 56 | 48 | 59 | 48 | 27 | 48 | 52 | 42 | 51 | 34 | 48 |
| CCT picture | 64 | 58.9 (3.1) | 46 | 39 | 56 | 23 | 41 | 30 | 26 | 46 | 43 | 32 | 37 | 30 | 23 | 20 | 19 | 28 | 26 | 34 | 17 | 28 |
| CCT word | 64 | 60.7 (2.06) | 52 | 53 | 56 | 31 | 53 | 44 | 45 | 40 | 43 | 46 | 42 | 47 | 16 | 18 | 25 | 47 | 39 | 47 | 21 | 28 |
| Environmental sounds | | | | | | | | | | | | | | | | | | | | | | |
| Spoken word – picture | 48 | 47.8 (0.6) | 48 | 39 | 47 | 30 | 43 | 40 | 40 | 40 | 44 | 32 | 26 | 40 | 26 | 21 | 23 | 40 | 32 | 30 | 32 | NT |
| Sound – picture | 48 | 41.2 (2.5) | 22 | 19 | 26 | 10 | 11 | 14 | 25 | 20 | 21 | 19 | 8 | 22 | 7 | 5 | 6 | 17 | 11 | NT | 10 | NT |
| Sound – word | 48 | 40.8 (3.8) | 25 | 13 | 25 | 11 | 14 | 12 | 21 | 14 | 21 | 15 | 10 | 13 | 11 | 5 | 11 | 15 | 15 | NT | NT | NT |
| Category fluency (6) | – | 95.7 (16.5) | 54 | 41 | 48 | 18 | 31 | 35 | 31 | 31 | 50 | 33 | 21 | 8 | 0 | 0 | 9 | 28 | 19 | 12 | 5 | 0 |
| Letter fluency | – | 44.2 (11.2) | 48 | 17 | 19 | 19 | 10 | 20 | 9 | 16 | 60 | 39 | 10 | 5 | 6 | 0 | 0 | 15 | 24 | 12 | 9 | 0 |

*denotes normal cut-off. NT = not tested. TA = Test abandoned.

strated these behavioural patterns previously for SD and SA patients, the results below concentrate specifically upon the new AD data.

### 3.3. *Indicators of deregulation*

#### 3.3.1. *Correlations between semantic and executive tasks*

For the AD groups, we assessed the relationship between performance on four executive tasks (elevator counting with/without distraction, WCST and Raven's matrices) and all ten tasks from the semantic battery (excluding letter fluency). The mild AD group exhibited significant associations between the elevator counting task and six semantic tasks, including: WPM, word and picture versions of the PPT and CCT as well as category fluency ($r > 0.56$, $p < 0.046$). Two further correlations approached significance (sound-picture and spoken word-picture matching from the environmental sounds battery: $r > 0.53$, $p < 0.059$). Significant associations were not observed for the remaining executive tasks, which is probably attributable to floor effects on these assessments. Notably, however, associations that approached significance were observed for the following task combinations: 1) elevator counting with distraction and word PPT/sound-picture matching ($r > 0.47$, $p < 0.087$); WCST and word PPT/word CCT/category fluency ($r > 0.47$, $p < 0.088$) and, 3) Raven's matrices and picture PPT/picture CCT/spoken word-picture matching ($r > 0.51$, $p < 0.068$).

The more severely impaired patients also exhibited a degree of association between their performance on semantic and executive tasks. Significant correlations occurred for the elevator counting task (without distraction) and the following semantic assessments: word PPT, both versions of the CCT and category fluency ($r > 0.55$, $p < 0.052$). Associations approaching significance were observed for elevator counting with distraction/word PPT and Ravens matrices/picture CCT ($r > 0.51$, $p < 0.067$).

#### 3.3.2. *Factors affecting performance on CCT*

Jefferies and Lambon Ralph [1] found that measures of the semantic control required to complete a task were able to predict item-by-item performance differentially in SA and SD patients. The ease with which distractor responses could be rejected (as rated by control participants) in the CCT was particularly sensitive to SA patients' control deficit. The patterns exhibited by the mild and severe AD groups reflected those observed previously in SA and SD respectively. Performance on the CCT (mean word/picture score) correlated with ease of rejecting distractors for the mild AD group ($r = 0.43$, $p < 0.0001$; two-tailed) but not the severe patients ($r = 0.20$, n.s.). Using simultaneous logistic regression, a combined analysis revealed a significant interaction between group and semantic control demands

Table 2
Breakdown of picture naming errors

| Error type | SA | Mild AD | Severe AD | SD |
|---|---|---|---|---|
| Semantic | 0.33 | 0.72 | 0.60 | 0.45 |
| Phonological | 0.11 | 0 | 0.06 | 0.02 |
| Perseveration | 0.15 | 0.08 | 0.07 | 0.11 |
| Omission | 0.32 | 0.14 | 0.2 | 0.37 |
| Unrelated | 0.10 | 0.06 | 0.07 | 0.05 |

All perseverative errors made by the AD groups and SD patients were also semantically related to the target.

(Wald = 6.52, $p$ = 0.011), which was indicative of a greater influence of this factor on the mild AD group's performance (ExpB: mild = 1.51; severe = 1.16).

### 3.3.3. Naming errors/Cueing

On the 64 set picture naming task all groups made mostly semantic and omission errors (see Table 2). The SA and SD groups exhibited no differences in the frequency of semantic, unrelated, perseverative or omission errors. The same pattern was observed when the mild and severe AD groups were compared; the only significant difference occurred for phonological errors, which constituted a greater proportion of errors for the severe AD group ($t$ (18) = 2.45, $p$ = 0.03).

SA patients' impoverished picture naming can be improved significantly with the provision of external support in the form of phonemic cues. In line with the highly consistent nature of semantic impairment in SD, however, very little difference is observed between spontaneous and cued naming scores [58]. This, coupled with the high omission rate in SD, is indicative of degradation of the core representations in the semantic repository.

### 3.3.4. Cued and miscued naming

In a repeated measures ANOVA comparing the mild and severely impaired AD groups, there were significant main effects of cue type (cue/miscue/no cue; F (2,36) = 25.49, $p < 0.0001$) and group (mild/severe; F (1,18) = 6.16, $p$ = 0.02) as well as a significant interaction between the two (F (2,36) = 5.49, $p$ = 0.008; see Fig. 1). The mild group performed significantly better than the severely impaired group in the cued and uncued conditions (cued: $t$ (18) = 3.01, $p$ = 0.008; uncued: $t$ (18) = 2.56, $p$ = 0.02) but no difference was observed for the miscued condition ($t$ (18) = 1.59, n.s.). For the mild patients significant differences were observed between all three conditions (cued > uncued > miscued: $t$ (9) > 3, $p < 0.015$) whereas the severe group did not show a significant difference between the uncued and miscued conditions (cued > uncued: $t$ (9) = 2.95, $p$ = 0.016; uncued = miscued: $t$ (9) < 1).

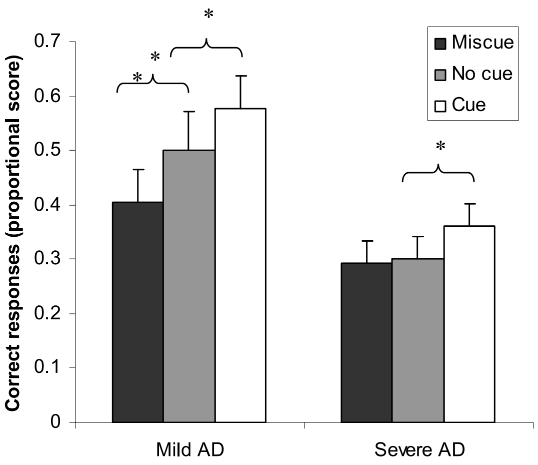

Fig. 1. Performance on the Boston Naming Test under different cue conditions. * indicates significant difference. Error bars denote standard error of the mean (SE).

### 3.3.5. Progressive phonological cueing

In a repeated measures ANOVA there were significant main effects of cue size (no cue/ two phonemes/ more than two phonemes provided: F (2,36) = 49.56, $p < 0.0001$) and group (mild/severe: F (1,18) = 6.60, $p$ = 0.019) but no interaction between the two (F (2,36) = 1.93, n.s.). We examined the proportion of phonemes required to cue an item that was not named spontaneously. On average the mild group required 37% the target's phonemes for successful cueing, which was substantially less than the severe group (56%; $t$ (17) = 3.33, $p$ = 0.004; patient BL was excluded from this analysis as no items were cued successfully).

## 3.4. Increasing indication of semantic degradation in severe AD

### 3.4.1. Naming and category fluency

The relative contribution of semantic control vs. representation varies across different semantic tasks and thus the relative performance across these tasks can be used to diagnose which aspect(s) of semantic cognition are affected in a particular disorder. We examined patients' performance across picture naming and category fluency. These tasks draw on knowledge from the same semantic categories but differ in important ways. Picture naming is fundamentally dependent on the integrity of a concept's representation. A degree of control is, arguably, also required to inhibit competing names. Like picture naming, category fluency requires intact semantic knowledge but also places substantial demand on control processes. Participants must perform an online search of the semantic repository, se-

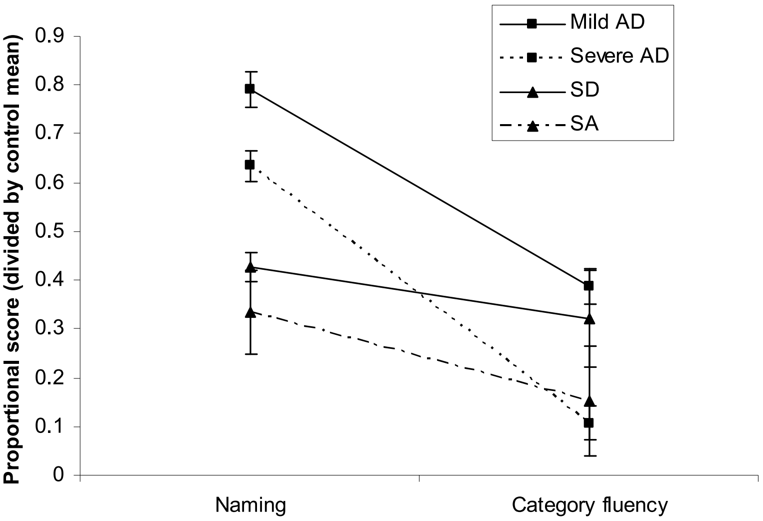

Fig. 2. Naming and category fluency performance. Verbal fluency tasks, unlike confrontational naming, do not have a maximum score. Thus to compare performance directly we expressed the patients' performance as a proportion of the control mean on each task. Error bars denote SE.

lecting items that possess key characteristics of a given category and rejecting close competitors, as well as monitoring previous responses. The demand on control processes becomes greater as representations degrade and normal category boundaries become distorted.

All groups performed within the impaired range for both tasks but the pattern across tasks differed across the groups (Table 1). While all four groups performed worse on the category fluency task compared to naming, this decline was exaggerated for the severe AD group (raw scores divided by control mean; see Fig. 2). In repeated measures ANOVAs, significant task by group interactions were found when the severely impaired AD patients were compared with each of the other patient groups (mild AD: F (1,18) = 5.39, $p = 0.032$; SA: F (1,17) = 9.14, $p = 0.008$; SD: F(1,18) = 6.27, $p = 0.022$). Borderline interactions were also observed when the mild AD group were compared with the SA patients (F (1,17) = 3.14, $p = 0.094$) and the SD group (F (1,18) = 3.06, $p = 0.098$). Performance on category fluency was significantly worse than naming in the severely impaired AD patients ($t$ (9) = 13.51, $p <$ 0.0001) and the mild AD patients ($t$ (9) = 10.33, $p <$ 0.0001), but not the remaining two groups (SA: $t$ (8) = 2.23, n.s.; SD: $t$ (9) = 0.64, n.s.).

These results are consistent with the view that there is a combination of regulatory control failure and degrading semantic representations in the later stages of AD. The milder patients showed a similar but less acute decline in performance across the two tasks, reflecting a gradual transformation from a purely semantic control disorder into a combined storage and control disorder across the course of the disease.

### 3.4.2. *Consistency*

Simultaneous logistic regression was used to determine whether items passed or failed in one task would predict performance on the same items in a different task. Familiarity was also included as a predictor because it contributes to some of the consistency exhibited by the SD patients. In addition to separate analyses of the different patient groups, combined analyses were used to compare consistency across the different groups. Patients who score at floor/ceiling on a task were excluded from the analysis (i.e., < 10% or > 90% correct). Previous studies found significant item consistency for SD and SA patients for within task comparisons but only consistency for the SD patients when comparing across different task types.

#### 3.4.2.1. *Consistency across different modalities within the same semantic task*

The mild and severe AD groups also performed consistently across word and picture versions of the CCT (Mild: $n = 10$, Wald > 26.15, $p < 0.0001$; Severe: $n = 9$, Wald < 16.02, $p < 0.0001$). Familiarity was a significant, or nearly significant, predictor of performance on both CCTp/CCTw tasks for the mild and severely impaired groups (Mild: Wald = 4.18 and 9.97, $p = 0.04$ and 0.002; Severe: Wald = 14.87 and 2.8, $p =< 0.0001$ and 0.094, CCTp and CCTw respectively). Interactive analyses did not reach significance (Wald < 1.05).

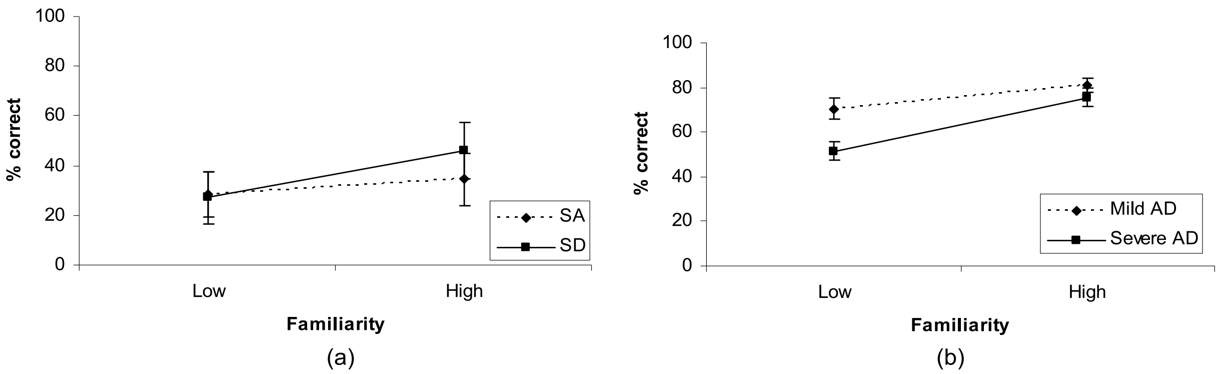

Fig. 3. Familiarity effects in picture naming. Error bars denote SE.

Consistency was also examined across three tasks from the environmental sounds battery: sound-picture, sound-word and spoken word-picture matching.

The mild AD cases exhibited consistency for 9/12 analyses (sound-picture/sound-word n = 10, spoken word-picture $n = 7$; Wald = 4.19–66.95, $p = 0.04 - < 0.0001$) with exceptions arising for combinations of sound-word and spoken word-picture matching (Wald $< 1$). The severe group were consistent across all twelve comparisons (sound-picture $n = 7$, sound-word $n = 6$, spoken word-picture $n = 9$; Wald = 7.84–19.75, $p = 0.005 - < 0.0001$). Interactions approached significance for three task pairs, which were indicative of a greater degree of consistency in the severely impaired group (spoken word-picture vs. sound-picture; sound-picture vs. spoken word-picture; sound-word vs. spoken word-picture: Wald = 2.88–3.72, $p = 0.09$–0.054, mean ExpB: Mild = 1.71; Severe = 3.96).

*3.4.2.2. Consistency across different semantic tasks*

The mild AD cases only exhibited consistency for one task pair (naming/WPM, $n = 5$, Wald $> 7.40$, $p < 0.007$). In contrast the severely impaired group performed highly consistently across 6/10 task combinations (naming/WPM: $n = 9$, Wald $> 6.71$, $p < 0.01$; naming/CCTp: $n = 10$, Wald $> 4.33$, $p < 0.04$; WPM/CCTw, $n = 8$, Wald $> 3.41$, $p < 0.065$).

*3.4.3. Familiarity*

Naming performance on the 20 most familiar items in the 64 set battery was compared with that of the 20 least familiar items ($n = 20$ in each set). The SD patients showed substantially larger effects of familiarity than the SA group. When the mild and severe AD groups were compared (see Fig. 3), a significant group by familiarity interaction was found (F (1,16) = 4.34, $p = 0.054$; see Fig. 3b). The mild group were more impaired when naming lower familiarity items ($t$ (8) = 2.8, $p = 0.023$) and a highly significant effect of familiarity was observed for the severely impaired AD group ($t$ (8) = 5.07, $p = 0.001$). While the two groups performed comparably on the high frequency items, the more severely impaired group performed significantly worse on the low frequency items ($t$ (18) = 2.99, $p = 0.008$).

## 4. Discussion

We extended Jefferies and Lambon Ralph's [1] case-series comparison of SA and SD to examine the nature of semantic impairment in AD. Both mild and severe AD patients performed below the normal level across a full range of semantic assessments. When patients at mild and more severe stages of the disease were contrasted directly, however, qualitative differences in the nature of their semantic impairments emerged. These findings suggest that the evolution of Alzheimer pathology over the course of the disease affects separate components of semantic cognition at different times. While a failure of control processes leads to semantic deregulation initially, by the later stage of the disease semantic representations themselves have also begun to degrade. Our results could explain why it has not previously been possible to determine the nature of semantic impairment in AD without explicitly considering the impact of disease severity.

In their original case-series comparative study, Jefferies and Lambon Ralph [1] identified an important link between the semantic impairment exhibited by patients with SA and their impaired executive control processes. In the present study, the same pattern of deregulated semantic cognition was also exhibited by mildly impaired AD patients. Like SA patients, the mild AD

group showed an association between performance on attentional/executive tasks and assessments of conceptual knowledge. The severely impaired group also exhibited a degree of correlation between semantic and executive measures but fewer reached significance. We specifically explored the relationship between performance on the CCT semantic association task and the cognitive demands of an individual trial. It was found that the ease with which distractor responses could be rejected only correlated with performance for the SA and mild AD patients suggesting that executive burden was particularly important for these groups. Moreover, we investigated the effect of manipulating the degree of external constraint provided in a picture naming task and found striking group differences. As previously observed in the context of SA [1,58], the mild AD group showed substantial improvement when provided with the first phoneme of the target while performance diminished when patients were miscued with the phoneme of a semantic competitor. Conversely, the severe AD group showed only a limited improvement with highly constraining cues and no effect of miscues. The parallel nature of semantic deficits observed in mild AD and SA suggests that, like SA patients, the mild AD cases performed poorly on semantic tasks due to a failure to control or shape their knowledge in a task appropriate fashion. A relative lack of correspondence between SA and severe AD, however, suggests that a qualitatively different impairment underlies poor performance on semantic tasks at a later stage of AD.

We examined the hypothesis that the executive deficit underlying semantic impairment in the early stages of AD not only worsens over time but is eventually joined by an additional, gradual degradation of the semantic repository itself. This view anticipates that later stage AD patients should be less comparable with SA cases and begin to exhibit characteristics of degraded knowledge already described in the context of SD. To investigate the nature of semantic impairment in severe AD we examined patients' performance across tasks that made different demands on individual components of semantic cognition: picture naming and category fluency. Both tasks depend on a large repository of conceptual knowledge. In addition, however, category fluency also places a much greater demand on control processes than does picture naming. In order to generate category members, a participant must be able to search the semantic repository, select appropriate exemplars and reject inappropriate items as well as monitor previous responses. While all four patient groups obtained lower scores in category fluency than picture naming, this difference was particularly exaggerated for the severely impaired AD group. The growing influence of semantic degradation over the course of AD was also reflected by the increasingly consistent nature of patients' impairments. While the mild patients performed most consistently across the same task presented in different modalities (i.e., when the task demands remained constant), the severely impaired group also exhibited a degree of consistency across tasks tapping the same concepts but in distinct ways (e.g., naming/CCT). The different patterns of consistency observed in mild and severe AD echoed those already observed in SA and SD respectively. Moreover, a growing tendency toward item consistency in later AD coincides with the view that representations are gradually but permanently degraded in this condition. In line with this hypothesis, we observed an influence of concept familiarity not only in SD but also in the severe AD patients. Conversely the SA and mild AD patients showed no difference in performance for low and high frequency concepts.

By directly comparing patients with mild and severe AD, we have demonstrated that the nature of semantic impairment is not stable but subject to qualitative changes over the course of the disease. Disruption of the frontal-temporoparietal-cingulate control network leads to deregulation of semantic cognition in the early stages of the disease. This problem not only persists and worsens over time but also becomes compounded by degradation of amodal semantic representations supported by the ATL. Our results represent a key advance in understanding the nature of semantic impairment in AD. We assert that it is no longer useful to characterise AD as either a storage deficit or an access/control disorder. Instead, research in this area should be directed toward tracing changes in the nature of semantic impairment with disease progression.

## Acknowledgements

We would like to thank all of the patients and their carers for their support of our studies. We are also grateful to Dr. Sarah Craig for identifying suitable cases for the study. This work was supported by an MRC-ESRC studentship (to F. Corbett), and an MRC grant (G0501632). We are grateful to Profs. John Hodges and Karalyn Patterson for continuing to share the original SD data with us.
